# Examining Infants’ Individuation of Others by Sociomoral Disposition

**DOI:** 10.3389/fpsyg.2019.01271

**Published:** 2019-05-31

**Authors:** Hernando Taborda-Osorio, Ashley B. Lyons, Erik W. Cheries

**Affiliations:** ^1^Department of Psychology, Pontificia Universidad Javeriana, Bogotá, Colombia; ^2^Department of Psychological and Brain Sciences, University of Massachusetts, Amherst, MA, United States

**Keywords:** infants, cognitive development, sociomoral dispositions, individuation, social cognition

## Abstract

Early on infants seem to represent social actions of others from a moral perspective, evaluating others’ dispositions as “mean” or “nice.” The current research examined whether or not 11-month-old infants represent these sociomoral dispositions as deep and identity-determining properties using an object individuation task. Infants were shown two identical looking characters emerging sequentially from behind a screen and engaging in two different sociomoral actions. By using a looking-time paradigm the results show an interaction effect between the baseline and test trials, showing that infants seem to represent two different characters involved in the event, disregarding their same external appearance. This effect was mainly apparent when infants witnessed a negative event first in test trials. Experiments 2 and 3 control for alternative explanations. In Experiment 2 infants failed to individuate two characters when they are shown two identical looking puppets. In Experiment 3 infants fail to represent two characters when social information was taken away from the show. We discuss the possibility that by the end of the first year of life infants might represent sociomoral dispositions as diagnostic of individual identity.

## Introduction

Moral judgment is a fundamental part of our daily social life. Our constant evaluation of others’ behavior – categorizing others’ actions as nice or mean, helpful or unhelpful – comprises a moral sense that is a continuous influence on the ways we choose to interact with others (Tomasello, 2016). Moreover, the propensity to automatically infer the social disposition of others appears to take root very early in development. By the end of their first year of life infants spontaneously represent the social actions of others as positive or negative (Premack and Premack, 1997), predict agents’ social preferences based on their past sociomoral interactions (Kuhlmeier et al., 2003), and evaluate others’ actions whereby they reject mean agents and choose to interact with nice ones (Hamlin et al., 2007). Such moral evaluations have been examined across a range of different scenarios and levels of difficulty (see Hamlin, 2013b for a review). For example, infants as young as 6 months of age who observe a puppet whose goal (such as reaching the top of a hill or opening a box) is assisted or thwarted by others, seemingly represent the agents involved in the interaction as possessing a nice (positive) or mean (negative) social disposition, respectively (e.g., Hamlin et al., 2007; Hamlin and Wynn, 2011; cf., Salvadori et al., 2015). Furthermore, infants’ evaluations are dependent on the goals (Hamlin and Baron, 2014), intentions (Hamlin, 2013a), and knowledge the characters possess when interacting with one another (Hamlin et al., 2013b), suggesting that these abilities comprise the essential foundation for a later-developing system of moral judgment (Wynn, 2008). Such a core capacity for social evaluation may be the result of an evolutionary adaptation to deal with other people in cooperative contexts (Tomasello and Vaish, 2013).

As these previous studies show, infants are capable of distinguishing agents by the sociomoral dispositions they display. However, no prior research has investigated how central these moral dispositions are for representing the identity of people over time. The current study aims to investigate whether infants represent an agent’s moral disposition as a deep and identity-determining property. Currently, it is an open question whether infants represent the sociomoral behaviors other engage in as fleeting actions that are subject to change from one moment to the next, or instead as relatively stable traits that constitute an important part of an agent’s individual identity. In other words, are infants biased to represent helpful and unhelpful actions as arising from different types of individuals?

Previous research show evidence of trait-based reasoning in older children and adults. For example, adults heavily weigh memories and personality traits when judging whether or not someone is the same person (Rips et al., 2006; Rips, 2011). Thus, psychological factors are more crucial for tracking peoples’ identity than external features, such as their face or bodily features (Brook, 2014). Similarly, preschool-aged children hold the belief that moral traits such as “niceness” and “meanness” are stable over time (Liu et al., 2007; Diesendruck and Lindenbaum, 2009; Boseovski, 2010), treat them as inductively powerful features rather than as mere transient behavioral properties (Heyman and Gelman, 2000), and use trait labels “mean” and “nice” to predict others mental states (Heyman and Gelman, 1999). For instance, young children predict that people labeled as “mean” will have more negative motives than “nice” people (Heyman and Gelman, 1999). Taken together, evidence suggests that older children represent sociomoral behavior as reflecting stable psychological dispositions that are inherently part of an individual’s identity and that help organize the social world in a more or less categorical manner. This may reflect, or perhaps help explain, a widespread practice in many cultures to tell children stories about well-defined good and evil characters (Bloom, 2013).

A powerful way to explore the developmental origins of this type of trait-based reasoning is by using a classic individuation task (e.g., Xu and Carey, 1996; Kingo and Krojgaard, 2011). In this experimental paradigm, infants are shown a situation, where 2 objects emerge sequentially from behind one or two screens separated by a gap. In the two screen condition infants as young as 4 months are able to use the differing spatiotemporal trajectories of the objects to represent that there must be two individuals in the event, a process called “individuation” (Spelke et al., 1995). By contrast, in order to successfully individuate two objects in the one screen condition, where the spatiotemporal properties of each object are ambiguous (i.e., both objects appear from behind the same screen), infants must rely upon their representations of other properties. Studies using this paradigm have determined that infants are capable of using featural information, such as an object’s shape, size, and pattern, from very early on (Wilcox, 1999), and functional and language-related differences between objects by about 10–12 months of age (Xu and Carey, 1996; Xu and Baker, 2005; Futo et al., 2010). Most strikingly, this paradigm has revealed that not all perceptually salient property differences are treated equally. Infants will respond to an event portraying two very different looking objects as containing just a single individual if they share some deeper or more intrinsic property such as their category membership (Xu et al., 2004), ontological kind (Bonatti et al., 2002; Surian and Caldi, 2010), or physical “insides” (Taborda-Osorio and Cheries, 2018). For example, while infants who observed an object displaying self-propelled motion and agentive features (e.g., a worm) and another that looked like a typical inanimate object (e.g., a box) represented two individuals in the scene, infants failed to individuate two very different looking entities that were agents (e.g., a bee and a worm; Surian and Caldi, 2010).

Just as individuation tasks have been used to identify the diagnostic criteria that underlie infants’ representations of objects, the current project uses this same strategy to determine whether infants represent an agent’s sociomoral behavior as a relatively stable and identity-determining property. Do infants represent sociomoral behaviors as fleeting actions that are subject to change from one moment to the next, or instead as stable traits that constitute an important part of an agent’s individual identity? We tested this by merging the classic object individuation task (Xu and Carey, 1996) with a recent demonstration of infants’ sociomoral evaluation (Hamlin and Wynn, 2011). Specifically, we tested whether infants would use the type of sociomoral behavior they observe to individuate the number of agents that exist in an event. In all three experiments reported here, 11-month-old infants witness a puppet struggling to open a box. In Experiment 1 two identical looking characters emerged sequentially from behind a screen and engaged in two *different* sociomoral actions toward the puppet, helping or hindering its goal of opening the box. In Experiment 2 infants observed the same task except that the two identical-looking characters appeared at different times to engage in *identical* rather than different sociomoral behaviors (two helping or hindering actions). Finally, Experiment 3 examined whether infants’ individuation judgments were primarily driven by characters engaging in two perceptually different actions that lacked any sociomoral content.

## Experiment 1

### Participants

Sixteen 11-month-old infants (8 female) participated in this experiment (*M* = 11 months, 13 days, SD = 5 days). This age group was selected based upon similar individuation studies using infants in the 10–12 month age range (e.g., Xu and Carey, 1996). All participants were healthy, full-term infants recruited from the Amherst, Massachusetts area. All study procedures were approved by the University of Massachusetts Internal Review Board and written informed consent was obtained from each of the parents. Eight additional infants participated but were excluded from analysis because of fussiness (2), experimental error (4) and parental interference (2).

### Materials

**FIGURE 1:**
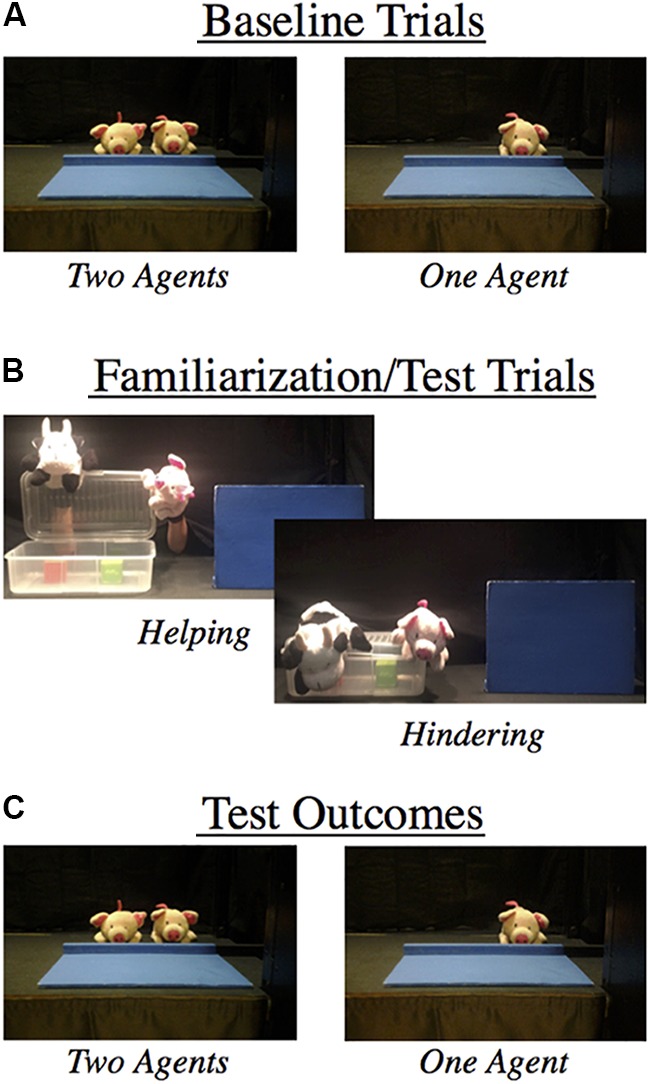
An outline of the experimental design depicting the **(A)** baseline trials, **(B)** familiarization/test events, and **(C)** test outcomes of Experiment 1.

Infants sat on their parent’s lap facing a black stage measuring 118 cm. wide × 75 cm. high. The room was dimly lit and parents were instructed to remain silent throughout the experiment. Infants observed a transparent box (35 cm. wide × 19 cm. deep and 12 cm. high) resting on the center of the stage with two different-colored cubes (5 cm × 5 cm) inside. At the right corner of the stage infants observed a blue screen (25 cm high × 36 cm wide) in a vertical position. There was a gap of 12 cm between the screen and the right frame of the stage and a gap of 17 cm between the screen and the box. Three different puppets were used in the experiment, all measuring 18 × 10 cm. A cow puppet served as the “Protagonist” who struggled to open the box. A pig puppet served as the “Opener” who emerged from behind the screen and helped the Protagonist to open the box by lifting the lid. Another identical pig puppet served as the “Closer” who hindered the Protagonist from opening the box by slamming the lid shut. A black curtain was lowered between trials to hide the stage. Two video cameras recorded events for posterior analyses, one focused on the infant’s face and the other focused on the stage (see Figure 1 for a depiction of the materials and stage display).

### Design and Procedure

Infants were shown 4 baseline trials, 2 familiarization trials, and 4 test trials in a typical violation-of-expectation design as described below (see Table 1 for the complete set of variables).

**Table 1:** A table depicting the counterbalance variables that were used across all three experiments.

Factor	Level 1	Level 2	Counterbalance Type
Sex	Male	Female	None
Trial Type	Baseline	Test	None
Test Action Order	Helper action first *(help, hinder; hinder, help)*	Hindering action first *(hinder, help; help, hinder)*	Within
Test Outcome	1 Object	2 Objects	Within
Outcome Order	1 Object first *(1, 2; 2,1)*	2 Objects first *(2,1; 1,2)*	Between

#### Baseline Trials

In the Baseline Trials, the curtain was raised revealing an upright blue screen on the stage, then one of the experimenters drew the infant’s attention to the stage using infant-directed speech (“Hi [baby’s name], look here”) before dropping the screen revealing either one or two identical pig puppets (see Figure 1). Infants’ looking time was recorded and the trial finished when they either looked away for at least two consecutive seconds or after 60 s of cumulative looking. This procedure was repeated for a total of 4 baseline trials. The number of revealed objects was counterbalanced across participants (baseline trial block: 1, 2, 2, 1 or 2, 1, 1, 2).

#### Familiarization Trials

The familiarization trials were modeled from the original box task used in previous demonstrations of infants’ moral evaluation that elicited reliable reaching preferences (Hamlin and Wynn, 2011). The familiarization trials were included to expose infants to the events and to help facilitate encoding of the sequence of actions that would be seen in the subsequent test phase. At the start of the event the Protagonist puppet entered the stage from the left corner and moved to one side of the box, which was positioned in the center of the stage. The puppet leaned down to look inside the box three times and then attempted to open the box four times by pulling on the corner of the box’s lid. On the first two attempts it pulled up, lifted the edge of the box a few inches, and dropped it back down. On the third and fourth attempts, it lifted the edge of the lid and lowered it while continuously holding onto the lid, as if the lid was too heavy for it to open. On the fifth attempt, a Pig puppet moved out from behind the opaque screen that was positioned on the right side of the stage, and moved forward next to the box. What happened next was determined by whether it was a Helping or Hindering trial.

During the Helping trial, the Pig puppet grasped the front right corner of the box, and both the Pig and Protagonist opened the box together. The Protagonist then reached into the box, retrieved one cube, and returned to its original location on the left side of the stage. The Pig closed the lid and returned back to its original position behind the opaque screen.

During the Hindering trial, the Pig puppet jumped on the corner of the box, slamming the lid closed. The Protagonist and Pig puppets then returned to their original locations (the left side of the stage and behind the opaque screen, respectively). Both Helping and Hindering trials lasted approximately 45 s. After the action on the stage had paused for 5 s the curtain was lowered and the trial ended. The order of these trials (Helping or Hindering trials first) was counterbalanced across participants.

#### Test Trials

Each test trial began by showing infants a full sequence of the same familiarization trial events (both a helping and hindering event) described above. In addition, test trial events included a second phase where, after each full helping/hindering sequence had ended, one of the experimenters drew the infant’s attention to the opaque screen on the stage (e.g., “Hi [baby’s name], look here”) and then dropped the opaque screen to reveal either 1 or 2 identical pig puppets resting on the stage. The number of puppets revealed behind the screen (1 or 2) and the order of the preceding events (Helping first or Hindering first) were both counterbalanced for each participant in two trial blocks (1, 2; 2, 1 or 2, 1; 1, 2, and Helping, Hindering; Hindering, Helping or Hindering, Helping; Helping, Hindering). The duration of the infants’ looking time was coded by two independent observers who were naive to the condition. The inter-observer agreement was high (*r* = 0.96).

### Results

**FIGURE 2:**
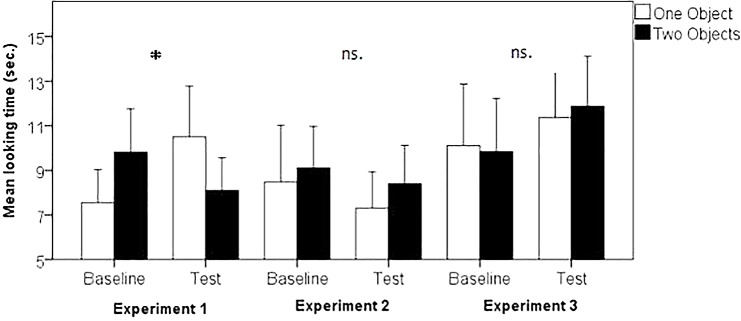
Looking times in Experiments 1–3, contrasting one vs. two objects as outcomes. Error bars represent standard error of the mean. Asterisks mark statistical significant differences (*p* < 0.05).

Preliminary analyses found no main effects of sex, Outcome Order (1 object or 2 objects first) or Trial Order (Helping first or Hindering first); therefore, these variables were collapsed in subsequent analyses. Following previous individuation studies with a within-subject design (e.g., Xu et al., 2004; Kingo and Krojgaard, 2011) the index of object individuation in this task is the statistical interaction in looking time to the two different object outcomes (1 vs. 2 objects) between the baseline and test phases. As such, a 2 (Object Outcome: 1 or 2 objects) × 2 (Trial Type: baseline or test) repeated measures analysis of variance (ANOVA) was conducted. This analysis revealed a significant interaction between Object Outcome and Trial Type, *F*(1, 15) = 13.4, *p* < 0.01, η^2^*p* = 0.47, which resulted from longer looking times toward two object outcomes (*M* = 9.81 s, SD = 3.89 s) than one object outcomes (*M* = 7.56 s, SD = 2.93 s) in the Baseline Trials, and longer looking times toward one object outcomes (*M* = 10.5 s, SD = 4.54 s) than two objects outcomes (*M* = 8.09 s, SD = 2.92 s) in the Test Trials (Figure 2). Planned comparison *t*-tests of one- versus two-object outcomes revealed a significant difference in the Baseline [*t*(15) = −3.2, *p* = 0.02 *d* = 0.8, two-tailed, Bonferroni corrected, 95% CI = −3.75, 0.75], but a non-significant difference in the Test Trials [*t*(15) = 1.85, *p* = 0.16, *d* = 0.62, two-tailed, Bonferroni corrected, 95% CI = −0.34, 5.2]. Additionally, the non-parametric analysis revealed that 12 out of 16 infants exhibited a larger preference for the one object outcome (*p* = 0.04, via a binomial test) in the test trials, while in the baseline trials only 3 infants had the same preference (*p* = 0.01, via a binomial test). The difference between both conditions was significant (*p* = 0.004, Fisher’s exact test). Overall, these results show that in the test trials infants overcame their preference for looking longer toward the two objects outcome, providing evidence of infants individuating two different agents behind the screen. However, the planned comparisons failed to provide this evidence in test trials only.

In order to get a better understanding as to why the results in test trials did not reach a significant difference we conducted a new set of analyses. Each participant witnessed two test pairs, counterbalancing Object Outcome and Trial Order; therefore, we conducted an ANOVA to detect possible differences across test pairs in infants’ looking time. A 2 (Test Pair: Hinder block first or Hinder block second) × 2 (Object Outcome: 1 or 2 objects) × 2 (Trial Order: Helping first or Hindering first) mixed-design ANOVA revealed a significant interaction between Trial Order and Object Outcome, *F*(1, 14) = 9.4, *p* < 0.01, η^2^*p* = 0.4. This interaction was followed up with planned *t*-tests between one and two objects outcome for Hindering first trial and for Helping first trials. This comparison showed a significant difference in the Hindering first condition, *t*(15) = 3.2, *p* = 0.01, *d* = 0.69, two-tailed, Bonferroni corrected, 95% CI = 1.8, 9.1 (*M*_OneObject_ = 13.4 s, SD_OneObject_ = 8.7; *M*_TwoObjects_ = 7.9 s, SD_TwoObjects_ = 4.2), but no for Helping first condition, *t*(15) = −0.39, *p* > 0.5, *d* = 0.12, two-tailed, Bonferroni corrected, 95% CI = −4.1, 2.8 (*M*_OneObject_ = 7.6 s, SD_OneObject_ = 3.1; *M*_TwoObjects_ = 8.2 s, SD_TwoObjects_ = 5.1). These findings show that the difference between one and two objects outcome showed up only in the test pair where infants witnessed the hinder action first, regardless of whether it was the first or the second block. The ANOVA also revealed significant interactions between Test Pair and Object Outcome, *F*(1, 14) = 4.95, *p* = 0.043, η^2^*p* = 0.26, and between Test Pair and Trial Type, *F*(1, 14) = 6.6, *p* = 0.02, η^2^*p* = 0.32. However, planned comparisons do not show significant differences across simple effects in either case, correcting for multiple comparisons. No other interactions or main effects were significant.

Additionally, we examined the nature of the reported result in this experiment further by including a Bayes factor analysis using a one-sample *t*-test on the baseline-test trial difference score. This resulted in a Bayes Factor that strongly favored the experimental hypothesis (Scaled-Information Bayes Factor = 26.4; Rouder et al., 2009).

### Discussion

Overall, the results of this experiment suggest that infants’ expectation about the number of individuals involved in the event was significantly affected by the different preceding actions they observed. By 11-months of age infants overcome their baseline preference for two objects, showing different patterns of looking time in baseline and test trials. This evidence of object individuation is striking since previous studies have demonstrated that infants at this age require the presence of contrasting physical properties (e.g., color or shape differences) in order to represent objects as separate individuals (e.g., Xu and Carey, 1996; Van de Walle et al., 2000). In contrast, the current study suggests that infants will infer the presence of two individuals if they observe two different sociomoral actions, despite both puppets involved in the helping-hindering interactions displaying the same surface properties. This pattern indicates that infants may have interpreted the different sociomoral actions as relatively stable behavioral dispositions that were diagnostic of their being 2 puppets involved in the event (e.g., one who helps and one who hinders).

Additionally, we found that infants’ individuation response appeared to be strongest when the first social interaction they observed was negative. That is, infants had a stronger expectation of there being two separate individuals involved in the events when first viewed a puppet hindering another’s goal before seeing a helpful event. This result could be an instance of the so-called “negativity bias” previously reported in the moral development literature, where negative events are better remembered and weighted than positive events (e.g., Hamlin et al., 2010; Hamlin and Baron, 2014). This effect may be due to negative events being perceived as more diagnostic of individual’s underlying disposition or because negative events are much more salient and have a deleterious effect on infants’ memory of the relatively weaker positive event.

While one interpretation of the current results is that infants used the different sociomoral behaviors they observed as criteria for agent individuation, an alternative possibility is that infants merely represented two individuals behind the screen based on the *number* of actions they observed, regardless of how these actions differed. Indeed, previous studies have reported that 6-month-olds are able to individuate and enumerate actions from continuous motion (Wynn, 1996; Sharon and Wynn, 1998). For example, when infants witness a sequence of 2 identical actions (jumps) they dishabituate when observing 3 actions, even if both sequences have the same duration (Sharon and Wynn, 1998). Therefore, an alternative explanation for the pattern of results we observed is that infants count 2 actions based on the sequence of events within each trial (one helping and one hindering event) and expect a correspondence between the number of actions and the number of puppets behind the screen, resulting in longer looking times for 1 object than for 2 objects outcome in the test trials. To test for this possibility a second experiment was run using the same box task but presenting 2 *identical* moral dispositions within each trial, two helping or two hindering actions. If infants in the first experiment individuated the puppets solely because of the number of actions or events, the pattern of results should be replicated in the second experiment.

## Experiment 2

### Participants

Sixteen 11-month-old infants (8 females) participated in this experiment (*M* = 11 months, 12 days, SD = 5 days). All infants were recruited from the Amherst, Massachusetts area, all study procedures were approved by the University of Massachusetts Internal Review Board and written informed consent was obtained from the parents. Four additional infants participated but were excluded from analysis because of fussiness (2) and experimental error (2).

### Materials, Design, and Procedure

The materials, design and procedure for the second experiment were the same for that of Experiment 1, except that both social actions infants witnessed were identical in the pattern of motion and in the moral disposition they displayed (both helping actions or both hindering actions), which was counterbalanced across participants. In order to be consistent regarding the number of cubes that infants observe in the box across test trials and across experiments, the hindering event started off with only one cube inside the box, and the helping event started off with three cubes inside the box. The result of two helping actions and two hindering actions was always one cube inside the box. The inter-observer agreement of this experiment was high (*r* = 0.95).

### Results

Preliminary analyses found no main effects of sex, Outcome Order (1 object or 2 objects first) or Trial Order (Opening first or Closing first); therefore, these variables were collapsed in subsequent analyses. A 2 (Object Outcome: 1 or 2 objects) × 2 (Trial Type: baseline or test) repeated measures analysis of variance (ANOVA) yielded no significant main effect for Object Outcome, *F*(1, 15) = 2.08, *p* = 0.17, η^2^*p* = 0.01, and Trial Type, *F*(1, 15) = 0.71, *p* = 0.41, η^2^*p* = 0.04. This analysis did not reveal a significant interaction between Object Outcome and Trial Type, *F*(1, 15) = 0.105, *p* = 0.75, η^2^*p* = 0.01. Infants spent the same time looking at the 1 and 2 object outcomes in both the baseline trials (*M* = 8.48, SD = 5.09; *M* = 9.1, SD = 3.72, for one object and two objects, respectively, *t*(15) = −0.69, *p* > 0.5, *d* = 0.17, two-tailed, Bonferroni corrected, 95% CI = −2.55, 1.3) and the test trials (*M* = 7.3, SD = 3.27; *M* = 8.4, SD = 3.44, for 1 object and 2 objects, respectively, *t*(15) = −1.1, *p* > 0.5, *d* = 0.28, two-tailed, Bonferroni corrected, 95% CI = −3.2, 1.0). A non-parametric analysis revealed that significantly more infants had a larger preference for the 2 objects outcome in test trials with 12 out of 16 infants showing this pattern (*p* = 0.04, via a binomial test), reversing the results of Experiment 1, while in the baseline trials 9 infants displayed a preference for the 2 objects outcome (*p* = 0.4, via a binomial test). The difference between both conditions was non-significant (*p* = 0.46, Fisher’s exact test). No interaction effects were found between Object Outcome, Trial Order and Test Pair.

Since the overall preference during baseline trials was different compared to Experiment 1 (where infants exhibited a significant preference for 2 objects, overall) we examined these trials in more detail in a subsequent analysis. A 2 (Object Outcome: one or two objects) × 2 (Trial Pair: first or second) ANOVA of just Baseline Trials revealed a significant interaction *F*(1, 15) = 4.55, *p* = 0.05, η^2^*p* = 0.23, resulting from a larger difference between one object and two object outcomes in the second pair (*M* = 6.2, SD = 5.4 and *M* = 8.7, SD = 7.1, respectively, *t*(15) = −2.9, *p* = 0.02, *d* = 0.36, two-tailed, Bonferroni corrected) than in the first pair (*M* = 11.4, SD = 6.9 and *M* = 10.3, SD = 4.5, respectively, *t*(15) = 0.75, *p* > 0.5, *d* = 0.19, two-tailed, Bonferroni corrected). Since the baseline preference found during this second pair of trials was more similar to what was observed in Experiment 1 and might be a more analogous test, we compared the results of this pair to both pairs of Test Trials, which yielded no significant interactions, *F*(1, 15) = 0.95, *p* = 0.35, η^2^*p* = 0.06, for the first pair and *F*(1, 15) = 0.88, *p* = 0.36, η^2^*p* = 0.06, for the second pair.

Additionally, we examined the nature of the reported null result in this experiment further by including a Bayes factor analysis using a one-sample *t*-test on the baseline-test trial difference score. This resulted in a Bayes Factor that favored the null (Scaled-Information Bayes Factor = 2.85; Rouder et al., 2009).

Finally, we compared results across experiments using a 2 (Outcome: 1 or 2 objects) × 2 (Trial Type: baseline or test) × 2 (Experiment Type: Experiment 1 or Experiment 2) analysis of variance (ANOVA), which yielded a significant three-way interaction among Outcome, Trial Type and Experiment Type, *F*(1, 30) = 7.02, *p* = 0.01, η^2^*p* = 0.19. This interaction suggests that the pattern of infants’ looking responses in Experiment 2 were significantly different from that of Experiment 1.

### Discussion

The results of Experiment 2 show that infants failed to individuate 2 agents behind the screen when the puppets they saw engaged in identical sociomoral actions. Infants who viewed two instances of an identical-looking puppet either help or hinder the protagonist’s goal of opening the box did not look longer at outcomes of either 1 or 2 individuals present behind the screen during test trials. In other words, viewing two discrete action events did not lead infants to expect two puppets behind the screen. This pattern of results stands in contrast to those we observed in Experiment 1, where infants’ looking preferences were significantly influenced by viewing two different sociomoral actions. Taken together these results suggest that infants’ successful individuation in Experiment 1 was not a purely numerical response based on them counting the number of discrete events. Infants across both experiments observed two events in each trial, but what seemed to affect infants’ expectations of the number of puppets involved in the event was whether they saw two *different* or two of the *same* sociomoral actions. These results are consistent with the interpretation that infants are biased to perceive a puppet that engages in a helpful sociomoral action as a different individual than one who engages in the opposite sociomoral event a moment later.

A second alternative explanation that may account for the successful individuation we observed in Experiment 1 is that infants’ representations are driven by differences in action type, regardless of whether those actions are social or not. For instance, our helping and hindering actions differed not only by their sociomoral disposition, but also by the types of motion and perceptual patterns that constitute those actions. For example, our hindering actions were characterized by a puppet pushing the box’s lid down, whereas in the helping actions the puppet lifted the lid up. Second, our helping and hindering actions also involved differences in the first order goals that the characters demonstrate across the event. Namely, during a hindering action the puppet demonstrates the intention to close the box, which could result in the representation of that agent as a “closer,” while during a helping action the intention of the puppet is to open the box, which could result in the representation of that agent as an “opener.” Either of these alternatives, or both together, could be driving the individuation effect observed in Experiment 1 without requiring any sensitivity to sociomoral interaction *per se*, among the different characters involved in the event. In other words, are different sociomoral actions treated the same as two different actions of any type, even those devoid of any social meaning? In order to address this question a third experiment was conducted to determine how infants would respond after observing two separate events showing a character opening a box and then an identical-looking character closing a box. Although the mechanics of these actions were perceptually identical to those in Experiments 1 and 2, they were rendered non-social in the current study by eliminating the protagonist from the event, thereby avoiding any interpretation of the events in terms of a social interaction or disposition. If infants’ agent individuation is driven by differences in motion and first-order goals, then we should observe a pattern of results similar to those in Experiment 1.

## Experiment 3

### Participants

Sixteen 11-month-old infants (8 females) participated in this experiment (*M* = 11 months, 10 days, SD = 4 days). All infants were recruited from the Amherst, Massachusetts area, with approval from the University’s Institutional Review Board, and written informed consent obtained from the parents. Three additional infants participated but were excluded from analysis because of fussiness (2) and experimental error (1).

### Materials, Design, and Procedure

The materials and design of the third experiment was the same for that of Experiment 1, except that in the Familiarization and Test Trials the Protagonist (the cow) and the cubes inside the box were removed from the show. The pattern of motion of both the Opening and the Closing actions was the same as the pattern of motion used in the Helping and Hindering events in the previous two experiments. During Opening trials, the Pig puppet jumped on the frontal right corner of the box, pulling up the lid completely backward. During Closing events, the Pig puppet grabbed the lid to close the box in a forward movement. A pause of about 6 s was used between both actions. The inter-observer agreement of this experiment was high (*r* = 0.96).

### Results

Preliminary analyses found no main effects for sex, Outcome Order (1 object or 2 objects first) or Trial Order (Opening first or Closing first); therefore, these variables were collapsed in subsequent analyses. A 2 (Object Outcome: 1 or 2 objects) × 2 (Trial Type: baseline or test) repeated measures analysis of variance (ANOVA) yielded no significant main effect for Object Outcome, *F*(1, 15) = 0.02, *p* = 0.9, η^2^*p* < 0.01, and Trial Type, *F*(1, 15) = 1.57, *p* = 0.23, η^2^*p* = 0.09, nor any significant interaction between Object Outcome and Trial Type, *F*(1, 15) = 0.13, *p* = 0.72, η^2^*p* < 0.01. Infants spent the same time looking at the 1 and 2 objects outcome in both the baseline trials (*M* = 10.01, SD = 5.52; *M* = 9.85, SD = 4.78, for one object and two objects, respectively, *t*(15) = 0.16, *p* > 0.5, *d* = 0.04, two-tailed, Bonferroni corrected, 95% CI = −3.2, 3.7) and the test trials (*M* = 11.36, SD = 4.97; *M* = 11.88, SD = 4.45, for 1 object and 2 objects, respectively, *t*(15) = −0.4, *p* > 0.5, *d* = 0.1, two-tailed, Bonferroni corrected, 95% CI = −3.3, 2.26). No interaction effects were found between Object Outcome, Trial Order and Test Pair. Non-parametric analysis revealed that only 7 out of 16 infants had a larger preference for the one object outcome in the test trials (*p* = 0.4, via a binomial test), while in the baseline trials 8 out of 16 infants preferred the one object outcome (*p* = 0.6, via a binomial test). The difference between both conditions was non-significant (*p* = 1, Fisher’s exact test). Additionally, we examined the nature of the reported null result in this experiment further by including a Bayes factor analysis using a one-sample *t*-test on the baseline-test trial difference score. This resulted in a Bayes Factor that favored the null (Scaled-Information Bayes Factor = 2.82; Rouder et al., 2009).

Finally, a 2 (Object Outcome: 1 or 2 objects) × 2 (Trial Type: baseline or test) × 2 (Experiment Type: Experiment 1 or Experiment 3) analysis of variance (ANOVA) yielded a significant three-way interaction among Outcome, Trial Type and Experiment Type, *F*(1, 30) = 4.74, *p* = 0.037, η^2^*p* = 0.14. This interaction suggests that the pattern of results in Experiment 3 is significantly different from that of Experiment 1.

### Discussion

The results of Experiment 3 reveal infants’ failure to individuate two agents behind the screen after observing two different but non-social actions. Infants who viewed a puppet emerge from behind a screen to open a box and then an identical-looking puppet emerge to engage in the opposite action of closing the box looked equally long at 1 and 2 object outcomes, suggesting that they did not clearly represent how many agents were involved in the event. In other words, events involving two discrete and opposite actions are not sufficient for driving infants’ individuation judgments. This lack of sensitivity is striking since these events involved the same exact actions and movements (opening and closing a box) as those observed in Experiment 1. This suggests that infants’ individuation judgments are not merely based upon observing actions that are perceptually distinct from one another.

This pattern also suggests that infants are not inferring the number of individuals in the event by representing the number of first-order goals they have attributed to the agents. For instance, infants in Experiment 1 might have attributed to an agent the goal of “opening” the box in one moment and the agent’s goal of “closing” the box in the next and used that as the basis of their individuation judgment. However, despite the first order goals of the agents being equated, infants exhibited significantly different patterns of looking in the current experiment compared to those in Experiment 1. Taken together these results suggest that infants’ successful individuation in Experiment 1 was not purely a response based on them counting perceptually discrete events or goal states.

## General Discussion

The current study utilized an individuation task to investigate whether 11-month-old infants use social dispositions to keep track of the agents’ individual identity. Experiment 1 found that when infants observe two different sociomoral actions, such as helping and hindering, their looking pattern is consistent with them having an expectation of two agents, despite the agents looking perceptually identical. By contrast, infants in Experiment 2 who observed two separate but identical sociomoral actions (either helping-helping or hindering-hindering), failed to individuate two agents, indicating that infants do not infer the number of agents involved in the event solely based on the number of discrete actions they had perceived. Likewise, in Experiment 3 infants fail to individuate two agents based on differences in motion or distinct first-order intentions (e.g., puppets who engage in opposite actions, closing and then opening a box) alone, despite these events being the same as those actions infants witnessed in Experiment 1. However, it is worth highlighting that we did not find a significant effect in the test phase of Experiment 1 across both Helping and Hindering trials. Significant differences in test trials were obtained only in the Hindering first condition. Although an interaction effect in Experiment 1 indicates a significant change in the patter of infants’ looking time, stronger evidence of an individuation effect of sociomoral dispositions should be collected in future studies. Ideally, this evidence should be collected by comparing experiments with similar group’s baseline preferences. However, together these three experiments support the possibility that by the end of the first year of life infants represent intentions with sociomoral content as diagnostic of individual identity. While other types of social interactions might be sufficiently salient to drive similar individuation judgments, the difference between helpful and harmful actions might be an especially meaningful distinction for establishing social preferences (e.g., see Hamlin, 2013b for a review) and for tracking identity early in life.

The suggestion that infants’ sociomoral evaluations govern their judgments about identity in the current work may be useful in explaining prior demonstrations of sociomoral evaluation in infants (Hamlin et al., 2007). In these studies, 9-month-olds pick the character who previously displayed a prosocial action. One interpretation of this result in light of the current findings is that infants’ choice of who to select or reject is informed by an underlying attribution they make about the agent’s sociomoral identity. Infants’ choice, even in a third-party context may be supported by the belief that an agent’s past behavior is indicative of how it normally behaves or how it might behave in the future. This idea has support from recent work showing that 14-month-old infants seemingly expect an agent who has acted in a helpful manner toward another (e.g., helping them climb a hill) to also distribute resources fairly in another context (Surian et al., 2018). Therefore, it seems that early on in development infants are able to reason about agents’ sociomoral behaviors as stable and identity-determining dispositions. However, other authors (Liu et al., 2007) have claimed that the origins of trait-based reasoning may come from an understanding of labels as referring to kinds. Labeling, and namely generic language, has been shown to promote essentialist beliefs in the social domain (Rhodes et al., 2012). Thus, it could be the case that the use of trait labels leads children to infer that sociomoral behaviors come from internal and stable dispositions. The current individuation study suggest, however, that at the onset of language acquisition infants have already a basic intuition connecting sociomoral behaviors to different individuals. Over development, and through labeling, children may get a deeper understanding of sociomoral dispositions and engage in a more sophisticated social reasoning. For instance, although preschoolers understand the stability of sociomoral traits over time (Diesendruck and Lindenbaum, 2009), not until 8–9 years of age children are able to make trait-consistent predictions based only on observed behavioral information (Rholes and Ruble, 1984). Additionally, the early ability to represent sociomoral behaviors as stable dispositions suggested in the current research is only one part of a mature trait-based reasoning. Representing sociomoral disposition as traits also implies making rich inductions about possible behaviors, emotions and attitudes in different scenarios, and thus it implies a wider sense of identity (Heyman and Gelman, 1999).

Although the current research suggest that for infants sociomoral disposition are diagnostic of individual identity it is less informative as to how precisely they represent the identity of sociomoral agents. For example, the representation of the identity of an animal is different from the identity of an artifact (Kelemen and Carey, 2007). Animals, as natural kinds, are represented as possessing an objective and intrinsic identity, whereas artifacts are represented as possessing a more contextual and graded identity (Estes, 2003; Rips, 2011). The current research cannot determine how strongly infants connect an agent’s sociomoral behavior to their identity, as both natural and artifact kinds have been shown to support object individuation in infancy (Xu et al., 2004; Futo et al., 2010).

There are at least two possible interpretations of the current results. First, infants may represent an agent’s sociomoral disposition as a stable trait that is indicative of its kind or category. In support of this view, a recent study demonstrated that 9-month-old cannot form graded representations of prosocial and antisocial dispositions in the same agent (Steckler et al., 2017). Over time, children may become more flexible and admit graded representations of moral dispositions. A second possibility is that infants represent moral dispositions as a more relative and contextual trait from the start. This interpretation would be necessary for infants to exhibit more complex social inferences, as people engage in different types of social relationships with different people and in diverse situations. Indeed, previous research has demonstrated that infants are able to take into account contextual factors when reasoning about social behavior. For instance, infants prefer to interact with prosocial over antisocial agents, but they also prefer antisocial agents who harm dissimilar others (Hamlin et al., 2013a). Thus, they know that being “mean” or “nice” depends on the previous history of the characters involved. Either way, the current research suggests, first, that infants are able to use abstract psychological information to individuate different moral agents, and second, that they prioritize second-order over first-order intentions in doing so. However, more research is necessary to clarify how infants connect moral disposition to agents’ identity.

The issue about agents’ identity is also related to the observed asymmetry between Hindering first trials versus Helping first trials in Experiment 1. Infants seemed to have a stronger representation of two individuals when they witnessed the negative event first. This result is instructive since it suggests that the valence of the events witnessed had an effect on the infants’ looking time, something that did not manifest in Experiment 3, where social information was removed. However, future research should clarify what the reason of this effect was. For instance, the timing of the Helping-Hindering sequence in each trial was unusually long compared to previous individuation experiments (45 s) and this might be particularly harmful in the Helping first trials where the less salient (and more expected) event was shown first. This raises the possibility of having a stronger individuation effect in a future study by presenting a shorter Helping-Hindering sequence. If the difference still remains, then this effect may be telling of a deeper asymmetry in the infants’ representation of sociomoral actions.

A related open question concerns the specificity of the underlying representations infants are using, both in the current experiments and in prior studies using a social evaluation paradigm. To our knowledge, no prior research has determined how specific or abstract infants’ representations of such social interactions are. For example, infants may represent sociomoral dispositions that are very specific and conservatively bound to the context or action type in which they were observed (e.g., “the agent helped open the box”). Alternatively, infants may represent the same action in a deeper, more abstract way that refers to a general type of disposition (e.g., “the agent is a helper”). The latter possibility would be indicative of infants possessing a “kind” representation for sociomoral actions, where they represent a variety of sociomoral dispositions of the same valence as belonging to the same category. We are agnostic as to what type of representation may have driven the effects reported here. However, the object individuation paradigm could provide insights related to this distinction in the future by testing whether infants represent two different sociomoral actions of the same valence (e.g., helping an agent open a box and helping an agent climb a hill) as diagnostic of one or two agents. A failure in individuating two agents in this case compared to success in a task that involves two different events of different valences (e.g., helping an agent open a box and hindering an agent’s goal of climbing a hill) might indicate infants’ representation of such sociomoral interactions in a more kind-based manner.

Some statistical concerns still remain in this study. Significant differences in baseline looking times were obtained only in the Experiment 1, while the test trials did not reach a significant difference between one and two-objects displays. Although the same group of infants were compared across baseline and test trials, thus controlling for individual differences, it would be worthwhile to replicate the results of Experiment 1 by using different procedures in the future.

Finally, this research might also help inform how the representation of agents differs from the representation of physical objects in the infants’ mind. Unlike inert physical objects, agents’ behavior is better explained by internal non-obvious properties, in such a way that infants’ suppose that animal-like agents are endowed with internal physical properties (Setoh et al., 2013). Similarly, social behavior is better explained by internal dispositions as they have more predictive power than first-order intentions. In a previous study (Taborda-Osorio and Cheries, 2018), infants were shown to individuate agents based on the perception of internal physical properties while in the current one infants individuate based on moral dispositions. Thus, it appears that infants may represent agents as possessing diverse causal powers, and they pick these properties as more identity-determining than external properties when pitted against each other. Future research might expand on these findings by examining whether sociomoral dispositions are attributed to an agent’s internal properties and whether infants might also individuate based on other social behaviors (e.g., dominance) and social membership.

## Ethics Statement

This study was carried out in accordance with the recommendations of the University of Massachusetts Amherst Human Subjects IRB. The protocol was approved by the University of Massachusetts Amherst Human Subjects IRB. All subjects gave written informed consent in accordance with the Declaration of Helsinki.

## Author Contributions

HT-O, AL, and EC conceived and planned the experiments. HT-O and AL carried out the experiments. HT-O and EC contributed to the analysis of the results and to the writing of the manuscript.

## Conflict of Interest Statement

The authors declare that the research was conducted in the absence of any commercial or financial relationships that could be construed as a potential conflict of interest.
